# An engineered anti-Oropouche virus human-murine chimeric immunoglobulin M is a viable substitute for positive human serum controls in diagnostic serology assays

**DOI:** 10.1371/journal.pntd.0014642

**Published:** 2026-08-10

**Authors:** Kerri L. Miazgowicz, Christin H. Goodman, Amanda E. Calvert

**Affiliations:** Division of Vector-borne Diseases, Centers for Disease Control and Prevention, Fort Collins, Colorado, United States of America; University of Central Florida, UNITED STATES OF AMERICA

## Abstract

Oropouche virus (OROV) is an arbovirus of concern due to its recent geographical expansion and association with teratogenic outcomes. Serodiagnosis of OROV infection relies on methods such as the immunoglobulin M antibody-capture enzyme-linked immunosorbent assay (MAC-ELISA) and the plaque reduction neutralization test (PRNT). For re-emerging viruses such as OROV, sourcing large quantities of sera from acutely infected human donors for use as positive-control assay material is a significant challenge and hinders diagnostic capacity. To overcome this, we engineered and produced OROV119-chIgM, a human-murine chimeric IgM (chIgM) that expresses the variable regions of the murine monoclonal antibody OROV119, reactive to the Gc protein of OROV, on a human IgM backbone. OROV119-chIgM was evaluated in PRNT and MAC-ELISA to determine its suitability as a positive control in each assay. In PRNT, OROV119-chIgM achieved neutralization equivalent to an OROV polyclonal mouse hyperimmune ascitic fluid (MHIAF). Further, OROV119-chIgM exhibited superior performance compared with OROV-positive human donor sera in a MAC-ELISA. Overall, OROV119-chIgM is an attractive alternative to human donor sera assay control material because of its long-term sustainability and consistent batch-to-batch potency. OROV119-chIgM enhances diagnostic capacity by ensuring laboratories are poised to respond rapidly to future OROV outbreaks.

## Author summary

Oropouche virus (OROV) is a human pathogen transmitted by biting midges. OROV-infected individuals can develop mild febrile illness or, less frequently, severe neurological dysfunction. OROV infection is associated with poor birth outcomes in some infected pregnant women. Diagnosis includes detecting anti-OROV antibodies in patient sera in the early convalescent phase of the disease. Conducting diagnostic tests requires appropriate controls to confirm accurate test performance. Diagnostic test availability is hindered by the need for serum from donors who have recently been exposed to OROV to serve as positive control material. We used standard molecular biology techniques to design a novel, sustainable reagent produced *in vitro* that can replace limited donor sera while providing equivalent or superior performance in diagnostic tests, improving serology assay standardization across laboratories. Our positive control reagent, OROV119-chIgM, supports the development and standardization of OROV diagnostic testing by providing a renewable source of positive control material. OROV119-chIgM enhances diagnostic capacity globally by ensuring laboratories are poised for rapid response to future OROV outbreaks.

## Introduction

The re-emergence of Oropouche virus (OROV) in 2023–2025 in the Americas was notable due to high case counts [1], geographical expansion beyond previous outbreak regions [2], and the identification of atypical disease manifestations, including possible teratogenic outcomes (reviewed in [3,4]). The overlap among other pathogenic agents that cause non-specific febrile symptoms circulating in the region emphasizes the need for a definitive diagnostic test for OROV. OROV infection exhibits transient viremia with 60% of patients experiencing relapsing fever within 10 days of first symptom onset; thus initial molecular detection methods need to be performed in a relatively short window within seven days of symptom onset [5,6]. In contrast, serological diagnostics rely on detecting virus-specific antibodies elicited shortly after infection that persist for weeks to months, depending on the immunoglobulin class [5,7].

Serological diagnostic methods for arboviruses, such as OROV, include immunohistochemistry [8], the human IgG-ELISA [9], human IgM capture ELISA (MAC-ELISA) [10–12], and the plaque reduction neutralization test (PRNT) [13]. The MAC-ELISA detects OROV-specific IgM antibodies, a likely indicator of recent infection. PRNT detects anti-OROV neutralizing antibodies and is highly virus-specific. However, PRNT is not widely performed in all laboratories because it requires infectious virus and dedicated cell culture facilities.

Serodiagnostic assays require positive and negative controls to ensure the validity of test performance (CLIA, 42 CFR 493.1256). For reemerging viruses such as OROV, sourcing large quantities of sera from previously infected human patients to use as controls and correcting for assay performance variation between serum donors is a significant challenge. Murine monoclonal antibodies (MAbs) are not suitable for use in the MAC-ELISA, as the assay capture antibody specifically binds to human Fc_µ_. Thus, an anti-OROV-specific neutralizing MAb with a human Fc_µ_ is an attractive alternative. To address the need for a sustainable and consistent source of positive control material for both OROV IgM and neutralization serodiagnosis, we engineered a human-murine chimeric immunoglobulin M (chIgM) that neutralizes OROV in PRNT and efficiently captures OROV viral antigen in MAC-ELISA, circumventing the need for positive human donor sera.

## Results

### Production of OROV-specific human-murine chimeric IgM

Murine hybridomas producing IgG antibodies recognizing OROV epitopes were generated by BALB/cJ inoculation with purified UV-inactivated OROV (FL-240023). Hybridoma clone OROV119, which produces potent neutralizing anti-OROV MAbs, was selected to engineer a human-murine chimeric IgM (chIgM) as positive control material for serodiagnostic assays. To construct the chIgM expression vector, the sequences of the heavy (Hv) and light (Lv) chains of the murine IgG OROV119 were determined by targeted amplification and cloned into a human IgM/kappa expression vector [14]. OROV119-chIgM was transiently expressed in human Expi293F cells, purified, and its performance was assessed in PRNT and MAC-ELISA.

### OROV119-chIgM retains neutralization activity against OROV

**Fig 1:**
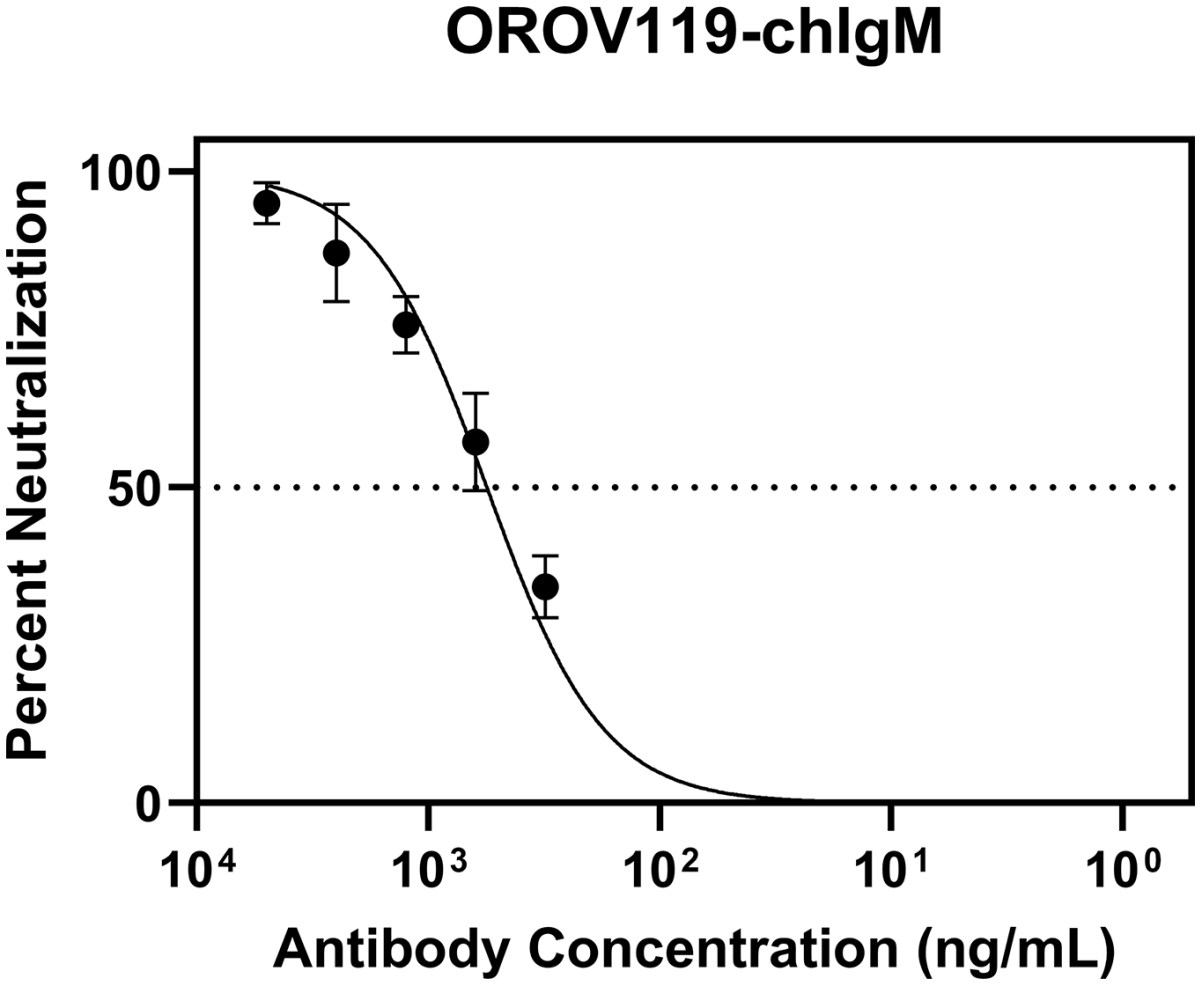
Neutralization reactivity of OROV119-chIgM with OROV (FL-240023). Percent neutralization of purified OROV119-chIgM was calculated based on virus input. A virus neutralization curve was generated as described in Materials and Methods. The dotted line represents 50% neutralization (IC_50_).

To assess neutralization activity of the engineered antibody, 2-fold dilutions of 5 µg/mL purified OROV119-chIgM were tested in triplicate in PRNT (Table 1). Dose-dependent neutralization was observed for both OROV119-chIgM and OROV MHIAF positive control polyclonal sera, but not for the negative-control sample, OROV106 MAb. Both neutralization potency metrics of OROV119-chIgM were consistent with a PRNT_50_ value of 625 ng/mL and an IC_50_ of 557.6 ng/mL (Table 1 and Fig 1). Almost five times the amount of chIgM is required to achieve 90% neutralization regardless of the metric. Overall, OROV119-chIgM achieved similar levels of neutralization as OROV-specific MHIAF polyclonal sera tested at 1:1000–1:4000 dilutions (Table 1).

**Table 1:** Evaluation of OROV119-chIgM in a PRNT.

Test Sera	Amount (ng/mL)	Percent Neutralization
OROV119-chIgM	5000	95
	2500	87
	1250	75
	625	57
	312.5	34
	156.25	17
	78.125	-14
	39.0625	-28
OROV MHIAF (positive control)	sera diluted 1:1000	96
	sera diluted 1:2000	87
	sera diluted 1:4000	64
OROV 106 (negative control)	5000	-18
	2500	-7
	1250	14

### OROV119-chIgM efficiently captures OROV antigen in a MAC-ELISA

In the MAC-ELISA, only molecules containing human Fc_µ_ are captured in the first step of the assay, allowing for discrimination of recent (IgM) and prior infections (IgG or other Ig classes). Thus, OROV119-chIgM can be used as a positive control in MAC-ELISA, as the antibody was engineered to contain human Fc_µ_ with OROV specificity conferred by the murine variable regions.

Compared to the positive human OROV IgM serum (laboratory-confirmed positive by diagnostic PRNT), OROV119-chIgM was more reactive at dilutions of 1:10–1:640 (chIgM: OD 1.919-1.035 vs. human sera: 0.667, along with respective P/N values) using concentrated transfection cell culture supernatant (Table 2). Furthermore, OROV119-chIgM exhibited less background reactivity to normal cell culture antigen across all dilutions tested between 1:10 and 1:5120 (OD: 0.005-0.008) compared to positive human serum (OD: 0.042) or negative human serum (OD: 0.016) tested at a 1:400 dilution, leading to higher negative background reactivity (NBR) values (Table 2) [15]. Overall, OROV119-chIgM exhibited improved MAC-ELISA assay performance characteristics at higher dilutions than the human positive control serum. Additionally, OROV119-chIgM showed high specificity to OROV, as no reactivity was detected with other viral antigens tested in the MAC-ELISA (Table 3).

**Table 2:** Evaluation of OROV119-chIgM in a MAC-ELISA.

Test Sera	Dilution	OD450 OROV Ag	OD450 Normal Ag	P/N^1^	NBR^2^	Interpretation^3^
OROV119-chIgM	1:10	1.919	0.006	95.96	302.24	POSITIVE
	1:20	2.016	0.007	100.81	294.34	
	1:40	1.916	0.007	95.82	275.73	
	1:80	1.811	0.005	90.53	344.89	
	1:160	1.797	0.006	89.86	323.81	
	1:320	1.647	0.006	82.34	291.46	
	1:640	1.035	0.008	51.75	130.18	
	1:1280	0.637	0.007	31.87	89.14	
	1:2560	0.318	0.006	15.88	52.50	
	1:5120	0.188	0.007	9.40	25.57	
human sera (positive control)	1:400	0.667	0.042	33.74	16.00	POSITIVE
human sera (negative control)	1:400	0.020	0.016	1.00	1.25	NEGATIVE

^1^P/N: Mean OD of the sample with OROV antigen (P)/ Mean OD of the negative control with OROV antigen (N).

^2^NBR (normal background ratio): Mean OD of the sample with OROV antigen/ Mean OD of the sample with normal antigen.

^3^Interpretation: Both P/N and NBR values are taken into consideration with interpretation defined as: POSITIVE (P/N ≥ 3.0 & NBR ≥ 2.0), EQUIVOCAL (P/N ≥ 2.0 OR < 3.0 & NBR ≥ 2.0), NEGATIVE (P/N < 2.0 & NBR any), and UNINTERPRETABLE (P/N ≥ 2.0 & NBR < 2.0).

**Table 3:** Cross-reactivity of OROV119-chIgM with viral antigen in the MAC-ELISA.

		Antigen Type										
		CHIKV	MAYV	VEEV	DENV	YFV	ZIKV	JCV	LACV	SSHV	OROV	Normal
Positive serum OD^1^		1.161	0.514	1.107	1.309	0.609	1.856	1.665	1.035	1.437	1.000	0.032^3^
Negative serum OD^2^		0.023	0.023	0.096	0.018	0.038	0.018	0.081	0.049	0.038	0.030	0.030
cIgM OROV119	1:100	0.014	0.017	0.056	0.007	0.021	0.012	0.021	0.026	0.020	2.047	0.012
	1:200	0.011	0.016	0.044	0.005	0.015	0.007	0.021	0.033	0.017	1.820	0.008
	1:400	0.012	0.014	0.044	0.006	0.013	0.008	0.022	0.028	0.020	1.430	0.008
	1:800	0.012	0.013	0.039	0.005	0.012	0.007	0.020	0.028	0.020	0.993	0.008
	1:1600	0.014	0.017	0.016	0.008	0.018	0.010	0.020	0.031	0.021	0.579	0.010
	1:3200	0.013	0.017	0.044	0.006	0.016	0.008	0.021	0.026	0.020	0.375	0.007

^1^OD (450 nm) of virus-specific positive serum dilutions to achieve an OD (450nm) reading between 0.5 and 2. Variations are based on the antigen being tested.

^2^OD (450 nm) of negative serum diluted 1:400 and tested on viral antigen.

^3^OD (450 nm) of OROV-positive serum diluted 1:400 tested on normal antigen.

## Discussion

A chimeric murine-human IgM monoclonal (OROV119-chIgM) was engineered to provide a consistent and sustainable source of positive control material for serological assays (PRNT and MAC-ELISA) detecting OROV-specific antibodies in human sera. Incorporation of OROV119-chIgM as positive control material in serological assays eliminates the need to acquire and consume limited human sera from OROV-infected individuals, as has been developed for several other arboviruses [10,16–19]. OROV119**-**chIgM potently neutralized a recent isolate of OROV (FL-240023) in PRNT and efficiently captured OROV viral antigen in a MAC-ELISA comparable to the limited pooled human sera control tested. By engineering OROV specificity onto a human Fc_µ_ molecule, OROV119-chIgM is suitable for use in the MAC-ELISA, which discriminates recent exposure from prior infection. OROV119-chIgM can be utilized in other serological applications compatible with human IgM molecules that include OROV Gc protein, such as IFA or flow cytometry.

The performance of an engineered monoclonal antibody (OROV119-chIgM) as positive control material was compared to polyclonal MHIAF in a PRNT or a limited pool of natural polyclonal OROV-positive human sera in a MAC-ELISA. In both assay formats, sera-derived antibodies are being tested for their ability to interact with assay-defined viral antigens. Thus, while the resilience of OROV119-chIgM to detect OROV epitopes across viral variants is currently unknown and warrants a thorough examination of the MAb epitope region, diagnostic assay performance is not substantially affected by natural viral evolution. During outbreak responses, it is infeasible to match the test viral antigen to current variants exactly because of the extensive performance characterizations that are mandated for each diagnostic test. Therefore, OROV119-chIgM would need to be tested against the selected antigen in each laboratory-developed test. Regardless, strong reactivity of OROV119-chIgM with the original 1955 OROV strain (TRVL-9760) viral antigen and its ability to potently neutralize a recent OROV strain from 2024 (FL-240023) implicates robust epitope conservation amid natural viral evolution through the most recent outbreak. The ability of OROV119-chIgM to bind future OROV variants is currently unknown but could be monitored and evaluated with genetic sequences from newly identified positive cases.

Work is currently ongoing to develop a cell line constitutively expressing OROV119-chIgM for continual production. While this work is in progress, we have shown that transient transfection can be relied upon to produce small batches of antibody, and production of OROV119-chIgM can be readily scalable to produce large quantities of antibody. For example, a single 50 mL small batch culture of OROV119-chIgM could support 83 PRNTs using 10 µg/mL stocks (PRNT_90_) or >100,000 MAC-ELISA tests using a 1:640 dilution (P/N 51.75). Thus, large production batches are suitable for extended use, as each liter of OROV119-chIgM culture can support >1,000 PRNT or >2,000,000 MAC-ELISA tests.

OROV119-chIgM was developed for use as a test control in diagnostic assays to confirm the assay was performed properly. Considerations for the implementation and use of OROV119-chIgM as assay positive control material should be made on a case-by-case basis. Each testing laboratory should thoroughly validate assays in accordance with their institutional and accrediting body regulations to ensure proper overall test performance. The long-term stability of OROV119-chIgM reactivity in various conditions is currently being evaluated for use at the CDC. Laboratories implementing OROV119-chIgM as positive test control material will need to evaluate it in their own testing environments based on their requirements.

OROV119-chIgM enhances laboratory capacity by providing a reagent that can be readily produced and distributed to laboratories to aid development and implementation of diagnostic serological assays with more consistent and defined positive control performance metrics. Overall, OROV119-chIgM helps ensure laboratories will be capable of responding rapidly to future OROV outbreaks by eliminating the need for limited and varied human sera for use as control material.

## Materials and methods

### Ethics statement

Ethics approval for use of previously collected human diagnostic specimens was obtained from the U.S. Centers for Disease Control’s (CDC’s) Human Subjects Institutional Review Board (CDC IRB #6773). Human specimens were delinked from any personal identifiers before the commencement of the study. Informed consent was not required in accordance with the CDC IRB determination because the study used previously collected, de-identified specimens.

Mice were handled as specified by the Division of Vector-Borne Diseases Institutional Animal Care and Use Committee recommendations (Protocol #25–001).

### Viruses and viral antigen

OROV isolate FL-240023 was acquired from the CDC Arbovirus Reference Collection (ARC) in Fort Collins, Colorado. Vero cells acquired from the CDC Arboviral Diagnostic Reagent Team (DRT) were maintained in DMEM supplemented with 5% FBS, pen/strep, and L-glutamine at 37°C and 5% CO2. Virus stocks were propagated once in Vero cells and supplemented with 20% FBS for storage. Virus purification was performed using a 30% glycerol-45% potassium tartrate gradient [20] and resuspended in 0.2M Tris (pH 8.0). Virus was inactivated using a UV Crosslinker (VWR) for 15 mins (585,000μJ/cm). Viral titers and inactivation were confirmed with one passage of virus in a standard plaque assay on Vero cells [21].

Viral and normal antigens for MAC-ELISAs were acquired from the CDC Arboviral Reference Collection. These antigens were derived from inactivated infected, VLPs, or uninfected cell-culture supernatants. All antigens were cell culture-derived except for VEEV antigen, which was derived from suckling mouse brain.

### Hybridoma generation

BALB/cJ (strain 000651) mice were purchased from The Jackson Laboratory and housed at the CDC animal facility in Fort Collins, CO, in accordance with CDC IACUC protocol #22–002. After a two-week acclimation period, mice were inoculated intraperitoneally four times, four to five weeks apart, with purified UV-inactivated OROV (FL-240023) mixed 1:1 with enhanced magic mouse adjuvant (CDN-A001E, Creative Diagnostics). The first inoculation contained 11 µg of virus, while subsequent inoculations contained 50 µg. Fusions were performed four days after the final boost using murine splenocytes with P3X63Ag8.632 murine myeloma cells following instructions in the ClonalCell HY Hybridoma cloning kit (03800, StemCell) and electrofusion in a 10mm gap BTX microslide using 39V alignment, with 2 pulses of AC: 30 sec and a 10 µs DC: 3000V. Hybridoma clone OROV119 was selected for chimerization based on its ability to neutralize OROV in a PRNT screen.

### OROV119-chIgM engineering and production

Signal sequences and heavy and light chain variable sequences of murine hybridoma OROV119 were determined by a murine IgG- and kappa-specific nested PCR using complementary DNA generated from messenger RNA, as previously described [22] (S1 Fig). PCR amplicons were sequenced using Oxford Nanopore sequencing (Plasmidsaurus). Human-murine chimeric IgM antibody (chIgM) was generated by PCR from a bicistronic mammalian expression vector, hIgM/kappa-pVITRO1 (61881, Addgene) [14] and combined with synthesized (Twist Bioscience) murine OROV119 heavy and light chain variable regions containing vector end compatibility using HiFi DNA Assembly 2X Master Mix (E5520S, NEB). Engineered plasmids were verified by full-length Oxford Nanopore sequencing (Plasmidsaurus) before transient transfection was performed using ExpiFectamine 293 Transfection Kit (A14524, ThermoFisher Scientific) with Expi293F cells (A14528, ThermoFisher Scientific) maintained in vented flasks shaken at 120 rpm at 37°C with 8% CO_2_. Transfection enhancers were added one day post-transfection, and cell supernatant was collected five days post-transfection. Cell-culture supernatant was concentrated using centrifugal filters (UFC701008, Millipore Sigma), followed by purification with the Pierce IgM purification kit (44897, ThermoFisher). Purified OROV119-chIgM concentrations were determined at 280nm using a NanoDrop OneC spectrophotometer.

### Plaque reduction neutralization test (PRNT)

To assess neutralization activity, purified OROV119-chIgM was tested in triplicate in PRNT. Approximately 75 PFU of OROV (FL-240023) were incubated with equal volumes of 2-fold dilutions of purified MAb starting at 10,000 ng/ml in BA1 (Hanks M-199 salts (M9163, Sigma-Aldrich), 0.05MTris pH 7.6 (15567027, Gibco), 1% BSA (81-066-4, Millipore), 0.35 g/L sodium bicarbonate (25080–094, Gibco), 100 U/mL penicillin, 100 mg/L streptomycin (15140–122, Gibco), 1 mg/L fungizone (SV30078.01, Hyclone) [19] for one hour at 37^o^C, 5% CO_2_. Anti-OROV MHIAF was used as a positive control (CDC ARC), and an anti-OROV murine IgG MAb without neutralization activity (OROV106) was used as a negative control. Six-well plates of Vero cells were inoculated with the virus-Ab complexes and incubated at 37°C, 5% CO_2_ for one hour after which cells were overlaid with 3 mL of nutrient agarose overlay (1% SeaKem LE agarose (50004, Lonza) in nutrient medium (0.165% lactalbumin hydrolysate (259962, VWR), 0.033% yeast extract (212750, ThermoFisher Scientific), Earle’s balanced salt solution, and 2% FBS) [16]. After incubation at 37°C, 5% CO_2_ for 48 hours, a second overlay containing 80 µg/ml of neutral red vital stain (091691149, MP Biomedicals) was applied. Plaques were counted 72 hours after infection, and percent neutralization was calculated based on input virus titer. PRNT_50_ and PRNT_90_ values were determined as the dilution of sera or concentration of MAb that achieved 50% or 90% reduction in input virus. A virus neutralization curve of OROV119-chIgM was generated by a 4-parameter non-linear regression dose-response and used to calculate the 50% inhibitory concentration (IC_50_) with the bottom and top of the curve constrained to 0 and 100, respectively (GraphPad Prism, v. 10) (S1 File).

### Human IgM capture ELISA (MAC-ELISA)

Concentrated OROV119-chIgM transfected cell culture supernatant was tested in MAC-ELISA as previously described [10]. Briefly, goat anti-human IgM (5210–0157, SeraCare) was diluted 1:2000 in carbonate/bicarbonate buffer, and coated plates were incubated overnight at 4˚C. Unbound antibody was removed, and nonspecific binding sites were blocked with 5% nonfat dry milk in PBS (0.5% Tween-20) for 30 minutes at room temperature. Plates were washed five times with PBS (0.1% Tween-20) wash buffer after each step in the assay, except for the wash after the conjugate step, where plates were washed ten times. OROV119-chIgM was serially diluted 2-fold in wash buffer, and OROV-IgM-positive and negative human samples were diluted 1:400 in wash buffer and added to the plate. For cross-reactivity testing, the following IgM positive serum control samples (CDC Human Subjects IRB protocol #6773) were diluted 1:150–1:3000 in wash buffer, depending on the viral antigen being tested, and added to the plate: chikungunya virus (CHIKV), Mayaro virus (MAYV), Venezuelan equine encephalitis virus (VEEV), dengue virus (DENV), yellow fever virus (YFV), Zika virus (ZIKV), Jamestown canyon virus (JCV), La Crosse virus (LACV), and Snowshoe hare virus (SSHV). After incubation of antibody at 37°C for one hour, OROV-, CHIKV, MAYV, VEEV, DENV, YFV, ZIKV, JCV, LACV, and SSHV positive or OROV-normal antigens were added and incubated overnight at 4˚C. Peroxidase-conjugated OROV MAb (for OROV and normal antigens), cross-reactive alphavirus MAb (for CHIKV, MAYV, VEEV antigens), cross-reactive flavivirus MAb (for DENV, YFV, ZIKV antigens), and cross-reactive orthobunyavirus MAb (for JCV, LACV, SSHV antigens) were incubated on the plate for one hour at 37°C. Enhanced K-blue TMB substrate (308175, Neogen) was added to each well, and plates were incubated in the dark at room temperature for 10 minutes. The reaction was stopped with TMB Stop solution (5150–0021, SeraCare), and plates were read at 450 nm (S2 File).

## Supporting information

S1 Fig Nucleotide and deduced amino acid sequences of MAb OROV119 variable regions. (A) variable region of the heavy chain. (B) variable region of the kappa chain. MAb OROV119-specific signal sequences are shown in aqua. Framework regions (FR) 1, 2, 3, and 4 are shown in yellow. Complementarity determining regions (CDR) 1, 2, and 3 are shown in blue.
(DOCX)

S1 File PRNT data used to calculate PRNT_50_, PRNT_90_ and IC_50_ of OROV119-chIgM. (XLSX)

S2 File (A) MAC-ELISA data generated to determine virus cross-reactivity of OROV119-chIgM with other arboviruses. (B) Table of reagents used in MAC-ELISA detailing source of the reagent, catalog number, and dilution used in the assay.
(XLSX)
