# Supplementary material for: An engineered anti-Oropouche virus human-murine chimeric immunoglobulin M is a viable substitute for positive human serum controls in diagnostic serology assays
Source: PLoS Negl Trop Dis. 2026 Aug 10;20(8):e0014642. doi: 10.1371/journal.pntd.0014642 (PMC13475966; doi:10.1371/journal.pntd.0014642)
Supplement: Supplementary file 2 [file pntd.0014642.s005.docx]

Response to Reviewers' Comments:

Reviewer #1: Major technical comments:

PRNT: In lines 102–105, the comparisons presented are not meaningful, as a purified monoclonal preparation cannot be directly compared to a polyclonal serum sample. A more appropriate approach would be to dilute the chimeric IgM (chIgM) in negative human serum to achieve concentrations comparable to those expected in clinical samples. A similar issue is present in the ELISA experiments. The chIgM is tested as unpurified supernatant and compared directly to human samples. This is not a like-for-like comparison. Using purified chIgM diluted in negative human serum would have provided more meaningful results. Therefore, the statement in lines 121–123 is not adequately supported, given the limitations of the experimental design.

Thank you to the reviewer for this comment. According to the WHO’s, “Guidelines for Plaque Reduction Neutralization Testing of Human Antibodies to Dengue Viruses”, the following controls need to be included in each test for a run to pass: uninfected cell monolayer, appropriate plaque counts per well as determined by back titration of the input virus, little or no reduction in plaque counts with negative serum control, appropriate PRNT titer of positive control antibody, and no serum toxicity observed with low test serum dilutions.

The chimeric IgM is meant as a control to ensure an individual test is set up and run correctly, not as a control of specimen quality or composition. Determining or comparing specimen quality or composition is indeed an important aspect however and would be addressed during a thorough test validation to ensure proper overall test performance. In the CDC’s diagnostic PRNT, which is set up similarly to the above guidelines using appropriate growth conditions for OROV, when human sera or mouse hyperimmune ascitic fluid is not available, virus-neutralizing monoclonal antibodies (either as hybridoma supernatant or purified antibody) are used as a positive control in the test. In this case, the antibody composition is less important than the ability to neutralize the virus in the assay. Additionally, using purified antibody in the test allows comparison of the control runs and antibody preparations to ensure that PRNT titers are similarly reproducible over time.

Likewise, concentrated cell culture supernatant of OROV119-chIgM tested in the MAC-ELISA is meant as a control to ensure the test is set up and run correctly and not a control of specimen quality or composition. Determining or comparing specimen quality or composition is again an important aspect that would be addressed during a thorough test validation to ensure overall MAC-ELISA test performance. The OROV MAC-ELISA was set up similar to the CDC ZIKA MAC-ELISA under FDA’s Emergency Use Authorization Guidelines. In this test, concentrated cell culture supernatant from flavivirus group reactive 6B6C-1-chIgM is used as the positive control.

Minor comments:

Ethics statement:

Please clarify whether the human positive serum was collected specifically for this study or obtained from previously published work. Additionally, indicate whether donors were laboratory-confirmed positive for OROV infection.

Human specimens used in this study were previously tested diagnostic specimens sent to CDC’s ADB diagnostic laboratory for testing from United States’ public health laboratories. Human OROV-positive samples used in this study were laboratory-confirmed positive by PRNT testing. This was included in lines 116-117.

Hybridoma generation: Please include details on: The amount of antigen used for immunization, The method of virus inactivation, The immunisation schedule (number of boosts, timing between immunisations, etc.)

We have included more information on the immunization schedule of mice and antigen used. Lines 228-234 have been amended to read, “BALB/cJ (strain 000651) mice were purchased from The Jackson Laboratory and housed at the CDC animal facility in Fort Collins, CO, in accordance with CDC IACUC protocol #22-002. After a two-week acclimation period, mice were inoculated intraperitoneally four times, four to five weeks apart, with purified UV-inactivated OROV (FL-240023) mixed 1:1 with enhanced magic mouse adjuvant (CDN-A001E, Creative Diagnostics). The first inoculation contained 11 µg of virus, while subsequent inoculations contained 50 µg.”

IgM capture ELISA: Clearly define the OROV antigen (OROV-Ag) and the normal antigen (Normal Ag) used in the assay.

This information has been added to the Viruses section of the Methods (now Viruses and Viral Antigen) in lines 223-226.

Serum dilution: Please justify the choice of a starting dilution of 1:400 for human serum samples. Was this due to limited sample volume or another reason?

The serum dilution of 1:400 is the standard dilution for all MAC-ELISAs in the ADB diagnostic laboratory. Martin *et. al.* (2000) found that using a 1:400 serum dilution offered a good correlation between true endpoint values, eliminating the need to perform endpoint titrations on samples.

Reviewer #2: The methodology is clearly laid out and follows the objectives of the study. However, there are a few concerns that should be addressed:

Line 91: were the sequences for the heavy and light chains deposited on Genbank or another repository?

The heavy and light variable regions have been included in the supplementary data.

Was the chimeric IgM tested against other orthobunyaviruses for specificity?

Thank you to the reviewer for this comment. We have included testing of OROV119-chIgM on several different arboviral antigens in the MAC-ELISA. These results are included in lines 126-127, and we have added Table 3 to summarize this data. Details of the MAC-ELISA to detect cross-reactivity are in the Methods, lines 292-303.

Line 206: Did you factor in extinction coefficient when determining the protein concentration on the nanodrop? Or was this measurement a generic approximation?

The measurement was a generic approximation using the default reference setting (1 Abs = 1 mg/ml).

Reviewer #1: Could the authors explain the rationale for selecting a kappa versus lambda light chain?

The human kappa light chain has been used previously by our lab to develop several murine-chimeric IgM antibodies as positive controls in MAC-ELISAs. There was no advantage or disadvantage in choosing kappa over lambda for antibody expression for our purpose, as the µ chain is the only antibody fragment necessary to bind the capture antibody in the test. The kappa chain is the most prominent light chain in the human antibody response, and it tends to remain monomeric. This may be more advantageous over the lambda chain, which can form dimers, thereby causing more steric hindrance for the capture antibody when binding the µ chain in the ELISA.

Reviewer #1: The manuscript by Miazgowicz et al. describes the production of a humanised chimeric IgM intended for use as a positive control in serological assays for Oropouche virus (OROV). My main concern with this work is conceptual. A single monoclonal antibody (mAb) is not truly commutable with a clinical sample and, importantly, may not be sufficiently resilient to viral variation. There is no characterisation of the epitope (e.g. epitope mapping), making it difficult to determine whether the antibody targets a conserved region of the virus. A polyclonal preparation, or a mixture of 3–4 monoclonal antibodies targeting different epitopes, would represent a more appropriate and robust alternative.

At a minimum, I would expect the authors to acknowledge and discuss this limitation in the Discussion section.

Thank you to the reviewer for pointing this out. We have added several more discussion points on the limitations of our study throughout the discussion, including lines 161-177, which discuss the limited number of human samples and viral variants tested, and in lines 187-195, which discuss considerations for labs implementing the chIgM in their test strategies. Also, as discussed previously, the chimeric IgM is meant as a control to ensure an individual test is set up and run correctly, not as a control of specimen quality or composition. Determining or comparing specimen quality or composition is indeed an important aspect however and would be addressed during a thorough test validation to ensure proper overall test performance.

Reviewer #2: Conclusions (Discussion) section was quite short and can be expanded in terms of limitations.

Thank you to the reviewer for pointing this out. We have added several more discussion points on the limitations of our study throughout the discussion, including lines 161-177, which discuss the limited number of human samples and viral variants tested, and in lines 187-195, which discuss considerations for labs implementing OROV119-chIgM in their test strategies.

Reviewer #1: Minor issues:

Line 62 (Ref 5): I was unable to find evidence supporting the statement regarding transient viremia in the cited reference. Please provide a more appropriate source.

Thank you to the reviewer for pointing this out. OROV, like other orthobunyaviruses, exhibits transient viremia; the virus and viral RNA are only detectable briefly in serum upon infection of the host. The reference used for this statement outlined experiments of OROV-infected non-human primates. Similar patterns are observed in human diagnostics; however, there have been cases in which patients with OROV infection have experienced relapsing fever. This point has been clarified in lines 61-63.

Line 67 (Refs 8–9): These are not suitable references for PRNT methodology. Both manuscripts cite other studies where PRNT was performed, but do not themselves describe the assay. Please replace with primary methodological references.

A reference with a description of the PRNT method performed with OROV has been included.

Line 73: Please clarify the meaning of “CLIA, 42 CFR…”. This appears to be a typographical or formatting error.

The typo has been corrected.

Reviewer #2: The study by Miazgowicz et al describes a recombinant, chimeric IgM mouse antibody that was humanized to work as a control in their MAC-ELISA. Although the chimeric antibody would serve useful as a control, the work presented in this manuscript reads more like a methodology paper and may be inappropriate for PLOS NTD - perhaps submission to PLOS One may be more suitable.

Thank you to the reviewer for this comment, and we appreciate their viewpoint. Our laboratory’s mission is to support arboviral diagnostics in the U.S. and across the globe through the development of novel reagents for serological diagnostic assays for new and emerging viruses. We provide reagents to public health laboratories at no cost for which no commercial assays exist through the CDC Arboviral Reference Collection. This manuscript is one part of a broader study to develop new reagents for serological diagnosis of OROV infections. While we have more information on this antibody and others, along with new recombinant antigens and assays, those studies are not completed. Our goal with this manuscript was to get this information disseminated as quickly as possible, so that other diagnostic laboratories, especially those affected by the epidemic, could request this reagent from the Collection for their own assay development and validation. We reason that publishing this manuscript in PLoS NTDs, an open-access journal read by the scientific community in our field of work, would help us do that.

Reviewer #3: Thank you so much for the opportunity to review this manuscript titled “An Engineered Anti-Oropouche Virus Human-Murine Chimeric Immunoglobulin M is a Viable Substitute for Positive Human Serum Controls in Diagnostic Serology Assays.” Overall, this is a technically sound, concise, methodologically clear, and timely diagnostic-development study with practical public health relevance, particularly given the recent emergence of Oropouche virus disease in the Americas. Please, find below my comments and suggestions for the authors.

The Discussion currently lacks a dedicated limitations paragraph.

We have added several more discussion points on the limitations of our study throughout the discussion, including lines 161-177and lines 187-195.

The following should be explicitly acknowledged:

1. Validation against only one OROV isolate.

This has been addressed in the discussion in lines 161-177.

2. Limited human serum comparator panel.

The chimeric IgM is intended as a control to ensure an individual test is set up and run correctly, not to assess the quality or composition of the specimen tested. This has been included in the discussion in lines 161-177.

3. Absence of cross-reactivity testing.

Cross-reactivity testing has been added. We have included testing of OROV119-chIgM on several different arboviral antigens in the MAC-ELISA. These results are included in lines 126-127, and we have added Table 3 to summarize these data. Details of the MAC-ELISA are included in the Methods, lines 289-303.

4. Lack of long-term stability data.

Considerations for implementation of OROV119-chIgM into the diagnostic testing environment has been added to the discussion in lines 187-195.

5. Use of transient rather than stable expression systems.

This point has been addressed in the discussion in lines 178-181.

6. No inter-laboratory reproducibility assessment.

Considerations for implementation of OROV119-chIgM into the diagnostic testing environment have been added to the discussion in lines 187-195.

7. No field validation in endemic settings.

Thank you to the reviewer for making this point. As this is a positive control to be implemented in individual laboratory-developed tests, each lab would need to undergo its own validation of its assay using the OROV119-chIgM as a test control antibody. Providing the positive control antibody for use is the first step in the development of LDTs.

8. Unknown performance across evolving OROV strains.

More discussion on evolving OROV strains and OROV119-chIgM performance has been addressed in lines 161-177.

9. Line 102: clarify whether PRNT50 and IC50 are derived independently or from the same regression model.

PRNT50 is an estimated endpoint based on the serum dilution in which 50% of the plaques from the input virus are present. The IC50 is a calculated endpoint where the data are fitted to a regression model. Typically, diagnostic labs calculate PRNT90 to determine whether a specimen is positive for anti-viral neutralizing antibodies, and thus, this is why it was included in the experiment. This is included in the Methods on lines 276-281.

10. Line 176: specify whether UV inactivation was validated by serial passage.

This information has been added on lines 221-222 of the Methods.

11. Line 241: “peroxidase-conjugated OROV MAb)” contains an apparent parenthesis typo.

The typo has been fixed.

12. Consistently use either “chIgM” or “cIgM”; both appear in the manuscript.

The nomenclature has been changed throughout to be consistent.
