# Supplementary material for: An engineered anti-Oropouche virus human-murine chimeric immunoglobulin M is a viable substitute for positive human serum controls in diagnostic serology assays
Source: PLoS Negl Trop Dis. 2026 Aug 10;20(8):e0014642. doi: 10.1371/journal.pntd.0014642 (PMC13475966; doi:10.1371/journal.pntd.0014642)
Supplement: Supplementary file 3 [file pntd.0014642.s001.docx]

A

B
