# Supplementary material for: An engineered anti-Oropouche virus human-murine chimeric immunoglobulin M is a viable substitute for positive human serum controls in diagnostic serology assays
Source: PLoS Negl Trop Dis. 2026 Aug 10;20(8):e0014642. doi: 10.1371/journal.pntd.0014642 (PMC13475966; doi:10.1371/journal.pntd.0014642)
Supplement: Peer review history

# Peer review: Decision Letter 0

**DOI:** 10.1371/journal.pntd.0014642.r001

**Authors:** Daniel Limonta, David Safronetz

22 Jun 2026

PNTD-D-26-00958

An Engineered Anti-Oropouche Virus Human-Murine Chimeric Immunoglobulin M is a Viable Substitute for Positive Human Serum Controls in Diagnostic Serology Assays

PLOS Neglected Tropical Diseases

Dear Dr. Calvert,

Alternative serological diagnostic approaches for Oropouche virus (OROV) infection have not been extensively evaluated during previous outbreaks. With the explosive spread in the Americas of the novel reasssortant of OROV recently reported in the Brazilian Amazon, further research aimed at improving serodiagnostic tools is warranted. In this study, Miazgowicz et al engineered and tested a human-murine chimeric IgM against the Gc protein of OROV in PRNT and MAC-ELISA to be used as a potential substitute of positive control sera.

Thank you for submitting your manuscript to PLOS Neglected Tropical Diseases. After careful consideration, we feel that it has merit but does not fully meet PLOS Neglected Tropical Diseases's publication criteria as it currently stands. Therefore, we invite you to submit a revised version of the manuscript that addresses the points raised during the review process. The submitted manuscript includes two tables in the main text. Addressing the reviewers’ comments may provide opportunities to expand the data presentation and discussion, ultimately enhancing the rigor and significance of the work.

Please submit your revised manuscript within by Aug 21 2026 11:59PM. If you will need more time than this to complete your revisions, please reply to this message or contact the journal office at plosntds@plos.org. When you're ready to submit your revision, log on to https://www.editorialmanager.com/pntd/ and select the 'Submissions Needing Revision' folder to locate your manuscript file.

Please include the following items when submitting your revised manuscript:

* A letter that responds to each point raised by the editor and reviewer(s). You should upload this letter as a separate file labeled 'Response to Reviewers'. This file does not need to include responses to any formatting updates and technical items listed in the 'Journal Requirements' section below.

* A marked-up copy of your manuscript that highlights changes made to the original version. You should upload this as a separate file labeled 'Revised Manuscript with Track Changes'.

* An unmarked version of your revised paper without tracked changes. You should upload this as a separate file labeled 'Manuscript'.

If you would like to make changes to your financial disclosure, competing interests statement, or data availability statement, please make these updates within the submission form at the time of resubmission. Guidelines for resubmitting your figure files are available below the reviewer comments at the end of this letter.

As the corresponding author, your ORCID iD is verified in the submission system and will appear in the published article. PLOS supports the use of ORCID, and we encourage all coauthors to register for an ORCID iD and use it as well. Please encourage your coauthors to verify their ORCID iD within the submission system before final acceptance, as unverified ORCID iDs will not appear in the published article. Only the individual author can complete the verification step; PLOS staff cannot verify ORCID iDs on behalf of authors.

We look forward to receiving your revised manuscript.

Kind regards,

Daniel Limonta, MD, PhD

Academic Editor

PLOS Neglected Tropical Diseases

David Safronetz

Section Editor

PLOS Neglected Tropical Diseases

Shaden Kamhawi

co-Editor-in-Chief

PLOS Neglected Tropical Diseases

orcid.org/0000-0003-4304-636XX

Paul Brindley

co-Editor-in-Chief

PLOS Neglected Tropical Diseases

orcid.org/0000-0003-1765-0002

**Additional Editor Comments (if provided):**

**Journal Requirements:**

If the reviewer comments include a recommendation to cite specific previously published works, please review and evaluate these publications to determine whether they are relevant and should be cited. There is no requirement to cite these works unless the editor has indicated otherwise.

1) Thank you for including an Ethics Statement for your study. Please include:

i) A statement that formal consent was obtained (must state whether verbal/written) OR the reason consent was not obtained (e.g. anonymity). NOTE: If child participants, the statement must declare that formal consent was obtained from the parent/guardian.].

2) When completing the data availability statement of the submission form, you indicated that you will make your data available on acceptance. We strongly recommend all authors decide on a data sharing plan before acceptance, as the process can be lengthy and hold up publication timelines. Please note that, though access restrictions are acceptable now, your entire data will need to be made freely accessible if your manuscript is accepted for publication. This policy applies to all data except where public deposition would breach compliance with the protocol approved by your research ethics board. If you are unable to adhere to our open data policy, please kindly revise your statement to explain your reasoning and we will seek the editor's input on an exemption. Please be assured that, once you have provided your new statement, the assessment of your exemption will not hold up the peer review process.

3) In the online submission form, you indicated that All relevant data from this study will be made available upon study completion.. All PLOS journals now require all data underlying the findings described in their manuscript to be freely available to other researchers, either

1. In a public repository

2. Within the manuscript itself

3. Uploaded as supplementary information.

This policy applies to all data except where public deposition would breach compliance with the protocol approved by your research ethics board. If your data cannot be made publicly available for ethical or legal reasons (e.g., public availability would compromise patient privacy), please explain your reasons by return email and your exemption request will be escalated to the editor for approval. Your exemption request will be handled independently and will not hold up the peer review process, but will need to be resolved should your manuscript be accepted for publication. One of the Editorial team will then be in touch if there are any issues.

4) We have amended your Competing Interest statement to comply with journal style. We kindly ask that you double check the statement and let us know if anything is incorrect.

**Reviewers' Comments:**

Reviewer's Responses to Questions

**Key Review Criteria Required for Acceptance?**

As you describe the new analyses required for acceptance, please consider the following:

**Methods**

-Are the objectives of the study clearly articulated with a clear testable hypothesis stated?

-Is the study design appropriate to address the stated objectives?

-Is the population clearly described and appropriate for the hypothesis being tested?

-Is the sample size sufficient to ensure adequate power to address the hypothesis being tested?

-Were correct statistical analysis used to support conclusions?

-Are there concerns about ethical or regulatory requirements being met?

Reviewer #1: Major technical comments:

PRNT: In lines 102–105, the comparisons presented are not meaningful, as a purified monoclonal preparation cannot be directly compared to a polyclonal serum sample.

A more appropriate approach would be to dilute the chimeric IgM (chIgM) in negative human serum to achieve concentrations comparable to those expected in clinical samples.

A similar issue is present in the ELISA experiments. The chIgM is tested as unpurified supernatant and compared directly to human samples. This is not a like-for-like comparison. Using purified chIgM diluted in negative human serum would have provided more meaningful results.

Therefore, the statement in lines 121–123 is not adequately supported, given the limitations of the experimental design.

Minor comments:

Ethics statement:

Please clarify whether the human positive serum was collected specifically for this study or obtained from previously published work. Additionally, indicate whether donors were laboratory-confirmed positive for OROV infection.

Hybridoma generation:

Please include details on:

The amount of antigen used for immunisation

The method of virus inactivation

The immunisation schedule (number of boosts, timing between immunisations, etc.)

IgM capture ELISA:

Clearly define the OROV antigen (OROV-Ag) and the normal antigen (Normal Ag) used in the assay.

Serum dilution:

Please justify the choice of a starting dilution of 1:400 for human serum samples. Was this due to limited sample volume or another reason?

Reviewer #2: The methodology is clearly laid out and follows the objectives of the study. However, there are a few concerns that should be addressed:

Line 91: were the sequences for the heavy and light chains deposited on Genbank or another repository?

Was the chimeric IgM tested against other orthobunyaviruses for specificity?

Line 206: Did you factor in extinction coefficient when determining the protein concentration on the nanodrop? Or was this measurement a generic approximation?

Reviewer #3: (No Response)

**********

**Results**

-Does the analysis presented match the analysis plan?

-Are the results clearly and completely presented?

-Are the figures (Tables, Images) of sufficient quality for clarity?

Reviewer #1: see major technical comments above

Q: Could the authors explain the rationale for selecting a kappa versus lambda light chain?

Reviewer #2: The results, figures and tables are presented clearly.

Reviewer #3: (No Response)

**********

**Conclusions**

-Are the conclusions supported by the data presented?

-Are the limitations of analysis clearly described?

-Do the authors discuss how these data can be helpful to advance our understanding of the topic under study?

-Is public health relevance addressed?

Reviewer #1: The manuscript by Miazgowicz et al. describes the production of a humanised chimeric IgM intended for use as a positive control in serological assays for Oropouche virus (OROV).

My main concern with this work is conceptual. A single monoclonal antibody (mAb) is not truly commutable with a clinical sample and, importantly, may not be sufficiently resilient to viral variation. There is no characterisation of the epitope (e.g. epitope mapping), making it difficult to determine whether the antibody targets a conserved region of the virus. A polyclonal preparation, or a mixture of 3–4 monoclonal antibodies targeting different epitopes, would represent a more appropriate and robust alternative.

At a minimum, I would expect the authors to acknowledge and discuss this limitation in the Discussion section.

Reviewer #2: Conclusions (Discussion) section was quite short and can be expanded in terms of limitations.

Reviewer #3: (No Response)

**********

**Editorial and Data Presentation Modifications?**

Use this section for editorial suggestions as well as relatively minor modifications of existing data that would enhance clarity. If the only modifications needed are minor and/or editorial, you may wish to recommend “Minor Revision” or “Accept”.

Reviewer #1: (No Response)

Reviewer #2: (No Response)

Reviewer #3: (No Response)

**********

**Summary and General Comments**

Use this section to provide overall comments, discuss strengths/weaknesses of the study, novelty, significance, general execution and scholarship. You may also include additional comments for the author, including concerns about dual publication, research ethics, or publication ethics. If requesting major revision, please articulate the new experiments that are needed.

Reviewer #1: Minor issues:

Line 62 (Ref 5):

I was unable to find evidence supporting the statement regarding transient viremia in the cited reference. Please provide a more appropriate source.

Line 67 (Refs 8–9):

These are not suitable references for PRNT methodology. Both manuscripts cite other studies where PRNT was performed, but do not themselves describe the assay. Please replace with primary methodological references.

Line 73:

Please clarify the meaning of “CLIA, 42 CFR…”. This appears to be a typographical or formatting error.

Reviewer #2: The study by Miazgowicz et al describes a recombinant, chimeric IgM mouse antibody that was humanized to work as a control in their MAC-ELISA. Although the chimeric antibody would serve useful as a control, the work presented in this manuscript reads more like a methodology paper and may be inappropriate for PLOS NTD - perhaps submission to PLOS One may be more suitable.

Reviewer #3: Thank you so much for the opportunity to review this manuscript titled “An Engineered Anti-Oropouche Virus Human-Murine Chimeric Immunoglobulin M is a Viable Substitute for Positive Human Serum Controls in Diagnostic Serology Assays.” Overall, this is a technically sound, concise, methodologically clear, and timely diagnostic-development study with practical public health relevance, particularly given the recent emergence of Oropouche virus disease in the Americas. Please, find below my comments and suggestions for the authors.

The Discussion currently lacks a dedicated limitations paragraph. The following should be explicitly acknowledged:

1. Validation against only one OROV isolate.

2. Limited human serum comparator panel.

3. Absence of cross-reactivity testing.

4. Lack of long-term stability data.

5. Use of transient rather than stable expression systems.

6. No inter-laboratory reproducibility assessment.

7. No field validation in endemic settings.

8. Unknown performance across evolving OROV strains.

9. Line 102: clarify whether PRNT50 and IC50 are derived independently or from the same regression model.

10. Line 176: specify whether UV inactivation was validated by serial passage.

11. Line 241: “peroxidase-conjugated OROV MAb)” contains an apparent parenthesis typo.

12. Consistently use either “chIgM” or “cIgM”; both appear in the manuscript.

**********

PLOS authors have the option to publish the peer review history of their article (what does this mean?). If published, this will include your full peer review and any attached files.

If you choose “no”, your identity will remain anonymous but your review may still be made public.

**Do you want your identity to be public for this peer review?**  For information about this choice, including consent withdrawal, please see our Privacy Policy.

Reviewer #1: No

Reviewer #2: No

Reviewer #3: No

[NOTE: If reviewer comments were submitted as an attachment file, they will be attached to this email and accessible via the submission site. Please log into your account, locate the manuscript record, and check for the action link "View Attachments". If this link does not appear, there are no attachment files.]

**Figure resubmission:**

**Reproducibility:**

To enhance the reproducibility of your results, we recommend that authors of applicable studies deposit laboratory protocols in protocols.io, where a protocol can be assigned its own identifier (DOI) such that it can be cited independently in the future. Additionally, PLOS ONE offers an option to publish peer-reviewed clinical study protocols. Read more information on sharing protocols at https://plos.org/protocols?utm_medium=editorial-email&utm_source=authorletters&utm_campaign=protocols

# Author comment: Author response to Decision Letter 1

**DOI:** 10.1371/journal.pntd.0014642.r002

24 Jul 2026

Attachment Submitted filename: PLoSNTDs_OROV_IgM_response to reviewers_final.docx

# Editor report: Decision Letter 1

**DOI:** 10.1371/journal.pntd.0014642.r003

**Authors:** Stephanie Seifert, Daniel Limonta

2 Aug 2026

Dear Dr. Calvert,

We are pleased to inform you that your manuscript 'An Engineered Anti-Oropouche Virus Human-Murine Chimeric Immunoglobulin M is a Viable Substitute for Positive Human Serum Controls in Diagnostic Serology Assays' has been provisionally accepted for publication in PLOS Neglected Tropical Diseases. We appreciate the authors' efforts in revising the manuscript. The responses provided adequately address the reviewers' recommendations and the manuscript has improved substantially.

Before your manuscript can be formally accepted you will need to complete some formatting changes, which you will receive in a follow up email. A member of our team will be in touch with a set of requests.

Please note that your manuscript will not be scheduled for publication until you have made the required changes, so a swift response is appreciated.

IMPORTANT: The editorial review process is now complete. PLOS will only permit corrections to spelling, formatting or significant scientific errors from this point onwards. Requests for major changes, or any which affect the scientific understanding of your work, will cause delays to the publication date of your manuscript.

Should you, your institution's press office or the journal office choose to press release your paper, you will automatically be opted out of early publication. We ask that you notify us now if you or your institution is planning to press release the article. All press must be co-ordinated with PLOS.

Thank you again for supporting Open Access publishing; we are looking forward to publishing your work in PLOS Neglected Tropical Diseases.

Best regards,

Daniel Limonta, MD, PhD

Academic Editor

PLOS Neglected Tropical Diseases

Stephanie Seifert

Section Editor

PLOS Neglected Tropical Diseases

Shaden Kamhawi

co-Editor-in-Chief

PLOS Neglected Tropical Diseases

orcid.org/0000-0003-4304-636XX

Paul Brindley

co-Editor-in-Chief

PLOS Neglected Tropical Diseases

orcid.org/0000-0003-1765-0002

***********************************************************

p.p1 {margin: 0.0px 0.0px 0.0px 0.0px; line-height: 16.0px; font: 14.0px Arial; color: #323333; -webkit-text-stroke: #323333}span.s1 {font-kerning: none

# Editor report: Acceptance letter

**DOI:** 10.1371/journal.pntd.0014642.r004

**Authors:** Stephanie Seifert, Daniel Limonta

Dear Dr. Calvert,

We are delighted to inform you that your manuscript, "An Engineered Anti-Oropouche Virus Human-Murine Chimeric Immunoglobulin M is a Viable Substitute for Positive Human Serum Controls in Diagnostic Serology Assays," has been formally accepted for publication in PLOS Neglected Tropical Diseases.

We have now passed your article onto the PLOS Production Department who will complete the rest of the publication process. All authors will receive a confirmation email upon publication.

The corresponding author will soon be receiving a typeset proof for review, to ensure errors have not been introduced during production. Please review the PDF proof of your manuscript carefully, as this is the last chance to correct any scientific or type-setting errors. Please note that major changes, or those which affect the scientific understanding of the work, will likely cause delays to the publication date of your manuscript. Note: Proofs for Front Matter articles (Editorial, Viewpoint, Symposium, Review, etc...) are generated on a different schedule and may not be made available as quickly.

Soon after your final files are uploaded, the early version of your manuscript will be published online unless you opted out of this process. The date of the early version will be your article's publication date. The final article will be published to the same URL, and all versions of the paper will be accessible to readers.

For Research Articles, you will receive an invoice from PLOS for your publication fee after your manuscript has reached the completed accept phase. If you receive an email requesting payment before acceptance or for any other service, this may be a phishing scheme. Learn how to identify phishing emails and protect your accounts at https://explore.plos.org/phishing.

Thank you again for supporting open-access publishing; we are looking forward to publishing your work in PLOS Neglected Tropical Diseases.

Best regards,

Shaden Kamhawi

co-Editor-in-Chief

PLOS Neglected Tropical Diseases

Paul Brindley

co-Editor-in-Chief

PLOS Neglected Tropical Diseases
